# Modulating Notochordal Differentiation of Human Induced Pluripotent Stem Cells Using Natural Nucleus Pulposus Tissue Matrix

**DOI:** 10.1371/journal.pone.0100885

**Published:** 2014-07-23

**Authors:** Yongxing Liu, Mohamed N. Rahaman, B. Sonny Bal

**Affiliations:** 1 Center for Bone and Tissue Repair and Regeneration, Department of Materials Science and Engineering, Missouri University of Science and Technology, Rolla, Missouri, United States of America; 2 Department of Orthopedic Surgery, School of Medicine, University of Missouri-Columbia, Columbia, Missouri, United States of America; INSERM U791, LIOAD, France

## Abstract

Human induced pluripotent stem cells (hiPSCs) can differentiate into notochordal cell (NC)-like cells when cultured in the presence of natural porcine nucleus pulposus (NP) tissue matrix. The method promises massive production of high-quality, functional cells to treat degenerative intervertebral discs (IVDs). Based on our previous work, we further examined the effect of cell-NP matrix contact and culture medium on the differentiation, and further assessed the functional differentiation ability of the generated NC-like. The study showed that direct contact between hiPSCs and NP matrix can promote the differentiation yield, whilst both the contact and non-contact cultures can generate functional NC-like cells. The generated NC-like cells are highly homogenous regarding the expression of notochordal marker genes. A culture medium containing a cocktail of growth factors (FGF, EGF, VEGF and IGF-1) also supported the notochordal differentiation in the presence of NP matrix. The NC-like cells showed excellent functional differentiation ability to generate NP-like tissue which was rich in aggrecan and collagen type II; and particularly, the proteoglycan to collagen content ratio was as high as 12.5–17.5 which represents a phenotype close to NP rather than hyaline cartilage. Collectively, the present study confirmed the effectiveness and flexibility of using natural NP tissue matrix to direct notochordal differentiation of hiPSCs, and the potential of using the generated NC-like cells for treating IVD degeneration.

## Introduction

Intervertebral discs (IVD) degeneration is a significant physical condition that is frequently related to the loss of cellularity and matrix degradation in nucleus pulposus (NP) tissue, which is followed by pathological cascades and eventually causing severe discogenic low back pain. [1], [2] Cell therapy is highly promising to restore the cellularity, biochemistry and biofunctionality of the degenerative NP tissue. [3], [4] Currently, the technique is restrained by the lack of available human cell sources. Notochordal cells (NCs) are the ideal for the transplantation purpose but scarcely present in adult human NP tissue.[5], [6] Neither autologous nor allogenic sources are available for research or future clinical applications. As a result, alternative cells have been investigated including mesenchymal stem cells and chondrocytes. Although successful in many aspects, these alternative cell sources have major limitations, such as inferior ability to produce native-like NP tissue and vulnerability to the challenging microenvironment of intervertebral discs. [7], [8].

In view of regenerative medicine, NCs and the terminally differentiated nucleus pulposus cells (NPCs) are both phenotypically correct and desirable for the purpose. NCs are more important since they can generate the NPCs and play pivot roles homeostasis of the NP tissue. [3], [4], [9] Also, NCs may potentially survive better in the harsh NP microenvironement upon transplantation which are generally highly challenging for other transplanted cells. [7], [8] [10] The first step to develop the therapy is to generate high-quality, functional NCs from enabling sources. HiPSCs hold great potential due to their pluripotency, abundance, and patient specificity.[11] However, no efficient methods of generating NCs from hiPSCs have been reported prior to our work. Generally, a single or a spectrum of growth factors and cytokines are required to direct lineage-specific differentiation of stem cells. A recent study used Activin A and consequently several other cytokines to induce mouse notochordal cells from mouse embryonic stem cells followed by cell sorting, the yield of which was only ∼1%.[12] Another study sorted a CD24^+^ subpopulation from spontaneously differentiated mouse embryoid body which showed notochordal characteristics; the yield reached 28% but poor expandability of the generated cells was observed.[13] To find a more efficient method, we exploited the modulating effect of a natural extracellular matrix to direct the notochordal differentiation. Natural porcine NP tissue contains a large population of NCs, which indicates a niche suitable for the maintaining of notochordal phenotype.[14], [15] We proposed such a natural environment may contain sufficient mediators to direct notochordal commitment of hiPSCs. Our preliminary study showed when hiPSCs were cultured together with the porcine NP matrix, they successfully acquired notochordal phenotype, which was evidenced by the remarkable up-regulation of typical notochordal genes including brachyury (T), cytokeratin-8 (CK-8), and cytokeratin-18 (CK-18), and the functional differentiation into NP phenotype evidenced by the expression of aggrecan and collagen type II.[16] Given the simplicity and effectiveness of the method, it is intriguing to further develop the technique towards the massive production of high quality NC-like cells for future translational research and therapeutic applications. Also it is highly intriguing to further investigate the differentiation ability of the NC-like cells. It is highly expected that the cells can generate a matrix with truly native-like biochemistry that characterized by a high proteoglycans: collagen ratio. The correct biochemistry is critical for the successful restoration of the biophysical functionality of NP tissue.

The present study was designed to address the concerns. Different culture conditions were examined in parallel and the differentiation outcomes were characterized and compared. The functional differentiation to generate NP tissue was characterized at both the transcript and protein levels, and the ECM biochemistry of the generated tissue was quantified. This study further demonstrated the efficacy and flexibility of the new method to generate NC-like cells from hiPSCs under the influence of porcine NP tissue, and it showed the high potential of the hiPSC-derived NC-like cells for the future regeneration of nucleus pulposus tissue.

## Materials and Methods

### Preparation of porcine NP tissue

Porcine NP tissue was prepared from the spines of juvenile pigs. The tissue were obtained from a local abattoir within 36 hours post-processing (Heinzt Processing, Cuba, MO). The usage of the porcine tissue was acknowledged and permitted by the mentioned entity. The discs were dissected in a sterile environment and the nucleus pulposus (NP) tissue was collected, briefly rinsed in 70% alcohol, and washed with sterile saline. Then the NP tissue was frozen at −80°C and freeze-dried overnight. (No living cells were detected in the freeze-dried material by Live/Dead analysis). The freeze-dried NP tissue became crispy and it was readily ground into powders. The ground powder was prepared in sterile environment and added directly to the differentiation culture medium; the powder from one disc was added to two wells of 6-well culture plates.

### hiPSC culture

The human induced pluripotent stem cell line HDFa-YK26 was purchased from the Stem Cells Core at the University of Connecticut Health Center (Farmington, CT).[17] The cells were routinely maintained on Matrigel (BD Biosciences, San Jose, CA) in mTeSR-1 maintenance medium (STEMCELL Technologies, Vancouver, CA). Medium was replenished daily. Cells were cultured in an incubator at 37°C in humidified atmosphere with 5% CO_2_. Cells were passaged every 3–5 days. At each passage, spent medium was removed, cells were rinsed using phosphate-buffered saline (PBS) and dissociated using Dispase (BD Biosciences).

### Generation of NC-like cells

**Figure 1:**
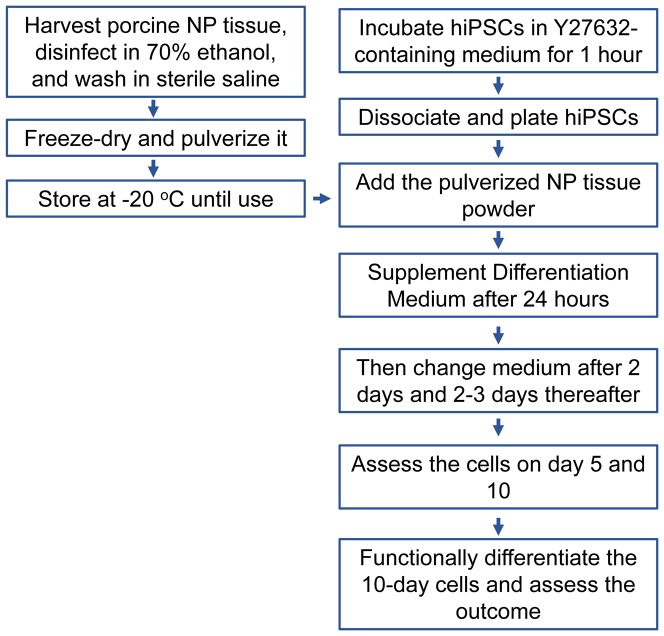
Workflow chart for the experiments.

**Figure 2:**
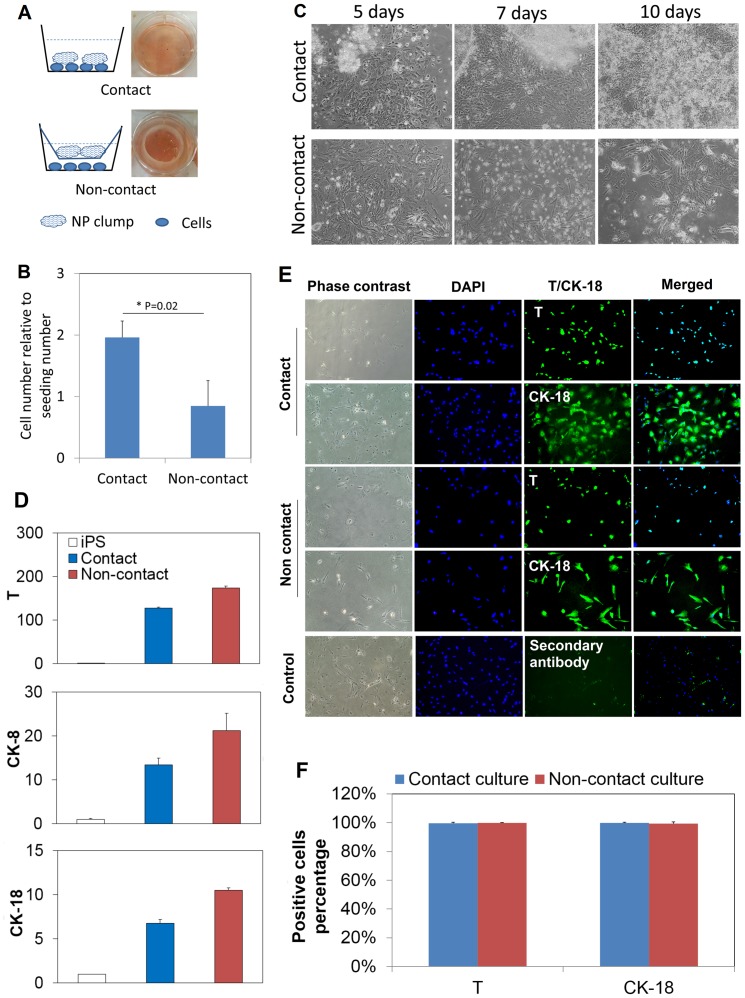
Notochordal differentiation of hiPSCs under the influence of porcine NP tissue matrix. (A): Illustration of the culture setups to allow or prevent direct contact between cells and porcine NP matrix. For the non-contact culture, the porcine NP tissue was placed in an insert with micro-openings that allow molecules transportation. Digital pictures of the cultures were shown on the right side of each schematic. (B): Cell numbers were counted after 10 days, which were reported relative to the initial plating numbers. The direct contact culture yielded 2 folds of numbers after 10 days which is remarkably higher than that of non-contact culture. *: p<0.05, n = 3. (C): Phase contrast images of cells in the culture wells. Some NP clumps became to attach to the culture well surface and came into contact with cells at approximately 7 days. Compact colonies were formed up to 10 days. Cells numbers appeared to increase over time in the contact culture. In contrast, many cells died at approximately 7 day, and much less cells were observed after 10 days. Magnification for all images: 100x. The observation is in good consistence with the quantification result. (D) Transcript of notochordal marker genes at day 10. Brachyury (T), cytokeratin-8 (CK-8) and cytokeratin-18 (CK-18) were all up regulated in both contact and non-contact cultures. The data are reported in relative mRNA expression which was analyzed by 2^−ΔΔCt^ method using undifferentiated hiPSCs as reference. Three biological samples were pooled and measured so that each result represents the average of three replicates. (E): Immunocytochemical staining images for the contact and non-contact cultures. Control group was omitted with primary antibody. DAPI: blue; T or CK-18: green. Magnification for all images: 100x. (F) Quantification of the positively stained cell numbers based on the immunocytochemical staining images. Total cells were counted based on DAPI staining. The reported results were averaged from four randomly selected images for each staining (n = 4).

The workflow is illustrated in Fig. 1. HiPSCs at passage 25 were used for the differentiation culture. Before dissociation, hiPSCs were incubated in the maintenance medium supplemented with 10 uM Y-27632 for 1 hour.[18], [19]_ENREF_21 The treatment is necessary for better survival of singly dissociated hiPSCs which are more responsive to exogenous stimulation.[19] HiPSC colonies were dissociated into single cells by incubating with Accutase (Millipore, Billerica, MA) for ∼10 min at 37°C and gently pipetting. The dissociated cells were spun down and re-suspended in the fresh maintenance medium supplemented with 10 uM Y27632 for plating. Approximately 150,000 cells suspending in 2 ml medium were plated in each well of 6-well culture plates. The pulverized NP tissue was added at the amount described earlier. The NP tissue was added either directly into each well (contact culture mode), or placed in an insert (70 um cell strainer; BD Bioscience) which was press-fitted in the culture wells; sufficient culture medium was added to the wells to cover the NP tissue in the insert (non-contact culture mode) (Fig. 2A).

After 24 hours, the culture medium was supplemented with an equal volume of differentiation medium. Two media was tested: Medium 1 contained alpha-minimum essential medium (α-MEM), 10% fetal bovine serum (FBS), 100 U/ml penicillin and 100 ug/ml streptomycin (all from Life Technologies, NY), while Medium 2 was a commercially available EGM-2-MV BulletKit (Lonza, Walkersville, MD) that contained 5% FBS and supplements of a cocktail of growth factors (FGF, EGF, VEGF and IGF-1). Medium 2 was routinely used in our laboratory to derive mesodermal lineage cells following a published method.[20], [21] Since the notochord has a mesoderm origin, this Medium 2 was expected to promote notochordal differentiation of hiPSCs too. The two-step procedure of changing medium is helpful to promote cell survival as tested in our previous studies.[19] Then, after two days, the medium was changed completely to the differentiation medium and replenished every two or three days thereafter. When changing the medium, avoid to withdraw the NP tissue out of the medium.

### In vitro generation of NP-like tissue

The NC-like cells harvested from the 10-day differentiation culture were further induced to differentiate and generate NP-like tissue in vitro. According to previously published studies, the conventional chondrogenic medium was suitable for the proposed differentiation. [15], [22], [23] Briefly, cells were pelleted in 5 ml polypropylene tubes (BD, NJ) by centrifugation of 200,000 cells at 400 g for 5 min. The differentiation medium contained high glucose Dulbecco's Modified Eagle Medium (DMEM) (Life Technologies), 1% ITS+ Premix (BD Bioscience), 100 mM sodium pyruvate (Life Technologies), 10 ng/ml TGF-beta 3 (Peprotech, NJ), 100 nM dexamethasone (Sigma-Aldrich, St. Louis, MO), 50 uM magnesium L-ascorbic acid-phosphate (Sigma-Aldrich), and 100 U/ml penicillin and 100 ug/ml streptomycin (Life Technologies). Medium was replenished every 2–3 days.

### Real time (RT)-PCR analysis

Total RNA was isolated using TRIzol and cDNA was synthesized using the High Capacity cDNA Reverse Transcription Kit (both from Life Technologies) following the manufactures' protocols. Real time PCR was performed on a Stratagene MX3000P QPCR system (Agilent Technologies, CA) using SYBR Green Master mix (Applied Biosystems, CA). For the notochordal differentiation culture, the following primers were used: brachyury (T) (Fwd: CAATGAGATGATCGTGACCAAGA; Rev: GCCAGACACGTTCACCTTCA), cytokeratin-8 (CK-8) (Fwd: CACATCTGTGGTGCTGTCCAT; Rev: GCCTTGACCTCAGCAATGATG), cytokeratin-18 (CK-18) (Fwd: GCCTACAAGCCCAGATTGC; Rev: GGCGAGGTCCTGAGATTTGG), and housekeeping gene beta-actin (ACTB) (Fwd: CGAGAAGATGACCCAGATCATG; Rev: ACAGCCTGGATAGCAACGTACA. [24] All primers were ordered from Realtimeprimers (Elkins Park, PA). Data were analyzed by 2^−ΔΔCt^ method using hiPSCs as reference. For the functional differentiation toward NP-like tissue, the following primers were used: aggrecan (ACAN) (Fwd: GTGCCTATCAGGACAAGGTCT; Rev: GATGCCTTTCACCACGACTTC), collagen type II (COL2A1) (Fwd: GGTCTTGGTGGAAACTTTGCT; Rev: GGTCCTTGCATTACTCCCAAC), collagen type I (COL1A1) (#VHPS-2103), Transcription factor SOX9 (#VHPS-8766) and housekeeping gene beta actin (ACTB) (Realtimeprimers Inc, Elkins Park, PA). Undifferentiated NC-like cells were used as reference for the analysis.

### Immunocytochemical analysis

The NC-like cells obtained from the 10-day differentiation cultures were examined for the protein level expression of T and CK-18. The cells were subcultured on a new culture plate and allowed to grow for one day in the differentiation medium. Before staining, the cells were fixed with 4% paraformaldehyde, permeabilized with 0.4% Triton X-100, and blocked in 1% bovine serum albumin (Sigma-Aldrich). Primary antibodies anti human T and Ck-18 (all from Santa Cruz Biotechnology, San Diego, CA) were added at 1∶200 dilution in staining buffer. After incubation at 37°C for 1 hour, the samples were rinsed three times with staining buffer. Then, FITC-conjugated secondary antibody (Molecular Probes, Eugene, OR) was added at 1∶500 dilution followed by incubation at 37°C for 1 hour, rinsing three times with the staining buffer. Cell nuclei were counter stained by DAPI (Sigma-Aldrich). The staining was visualized on a fluorescence microscope (Olympus BX53F, Center Valley, PA) equipped with a digital camera (Olympus DP70, Center Valley, PA).

### Immunohistochemical analysis

The 4-week pellet cultures were fixed in 10% neutral buffered formalin. Paraffin embedding was performed and 5 um sections were prepared using a microtome. Safranin-O staining was conducted routinely. For immunohistochemical analysis, sections were treated for 30 min at 37°C in 0.05% Trypsin (Life Technologies, NY). Endogenous peroxidase activity was quenched by incubation with 3% hydrogen peroxide (Sigma-Aldrich) for 10 minutes at room temperature. Primary antibodies raised against human aggrecan (cat# sc-16492) and collagen type II (cat# sc-28887) (both from Santa Cruz Biotechnology) were added at 1∶50 dilution and incubated for 1 hour at 37°C. After rinsing in PBS, biotin conjugated secondary antibodies recognizing immunoglobulin of rabbit (Cat#111-065-003) (Jackson ImmunoResearch Laboratories Inc, West Grove, PA) was added at 1∶1000 dilution and incubated for 1 hour at 37°C. After rinsing in PBS, streptavidin-conjugated horseradish peroxidase from an ultra-sensitive ABC peroxidase staining kit (Pierce, Rockford, IL) was added to react with the biotin conjugated secondary antibody for 30 minutes at 37°C, then rinsed, and a diaminobenzidine chromogenic substrate kit (Pierce, Rockford, IL) was used to develop the staining. Stained samples were examined in a transmitted light microscope (Olympus BX53F, Center Valley, PA) equipped with a digital camera (Olympus DP70, Center Valley, PA).

### Sulfated glycosaminoglycans (GAGs) and hydroxyproline content assay

Each pellet was digested for 24 hours at 56°C in papain buffer containing 3% papain, 5 mM L-cysteine, 50 mM EDTA and 0.1 M sodium acetate (all from Sigma-Aldrich), and the buffer was adjusted to a pH of 5.5. The amount of sulfated glycosaminoglycans (GAGs) was determined in an aliquot (20 µl) of the papain digest by 180 ul of dimethylene blue staining using chondroitin-4-sulfate (Sigma-Aldrich) as a standard on a microplate reader (FLUOstar Optima; BMG Labtech GmbH, Germany).

Hydroxyproline was measured following a method described previously.[25] Aliquots (80 ul) of each digest were hydrolyzed in 80 ul HCl (∼10 M) in screw-capped 1.5 ml polypropylene tubes (Fisher Scientific, IL) at 105°C for 16 hours. The hydrolysates were neutralized with 70 ul sodium hydroxide (10 M) and centrifuged to sediment particulate debris. Hydroxyproline (Sigma-Aldrich) standard solutions at concentrations of 2, 4, 6, 8, 10, and 12 ug/ml were prepared in 10 mM HCl. Aliquots (50 ul) of the hydrolysates and standards were placed in 96-well plates, followed by adding 100 ul oxidizing buffer supplemented with 3 wt/vol % chloramine T, and then adding 100 ul Ehrlich's reagent. The oxidizing buffer was prepared by mixing 6 ml isopropyl alcohol, 3.3 ml distilled water and 3.9 ml citrate-acetate buffer (containing 3.4 g sodium hydroxide, 3.4 g citric acid monohydrate, and 12 g sodium acetate trihydrate, and appropriate amounts of glacial acetic acid and water to made up a final volume of 100 ml with a pH of 6.0). The Ehrlich's reagent contained 6 g p-dimethylaminobenzaldehyde (p-DAB), 52 ml isopropyl alcohol and 16 ml of 50% perchloric acid (all from Sigma-Aldrich). After mixing the reagents thoroughly, the plate was covered with an adhesive seal and incubated for 45 min in a water bath at 60°C. Absorbance was read at 570 nm using the microplate reader mentioned above.

Both GAGs and hydroxyproline contents were normalized to dsDNA content, which was measured using PicoGreen assay (Molecular Probe, Gene, OR) following the manufacturer's manual. Fluorescence was measured at excitation of 350 nm and emission of 455 nm on the microplate reader mentioned above. dsDNA content was determined by comparing the readings with dsDNA standards.

### Statistical analysis

Student's t-test was used for the statistical analysis with the significance level set at 0.05.

## Results

### Generation of NC-like cells in a contact versus non-contact culture mode

The pulverized porcine NP matrix was added to the culture medium either directly or via an insert which allows the matrix to contact with the hiPSCs or not (Fig. 2A). After the freeze-dried NP tissue was added into the culture medium, it rewetted readily and formed gel-like clumps suspending in the medium. The plated cells did not attach to the tissue culture plate surface until supplementation of the serum-containing differentiation medium (was done on the second day). The contact or non-contact culture modes did not apparently affect the cell attachment process or cell viability in the first 5–6 days. At approximately 7 days, many of the NP tissue clumps began to attach to the cell layers in the contact-mode culture and cells seemed to grow robustly up to 10 days. Interesting, cells formed compact colonies associating the attached NP matrix (Fig. 2C). In comparison, many cells began to die at approximately 7 day, and the cell population did not show apparent expansion after 10 days in the non-contact culture (Fig. 2C). Quantification of the cell number clearly showed the difference; when compared to the initial seeding number, the cell number approximately doubled in the contact culture whereas increased little in the non-contact culture (Fig. 2B).

Transcripts of three notochordal marker genes were examined by RT-PCR (Fig. 2D). The cells remarkably expressed T, CK-8 and CK-18 genes comparing to the undifferentiated hiPSCs (Fig. 2D) in both contact and non-contact cultures. Note that all gene expressions were measured from a pool of three biological replicates, so they provide a good representation of the average level of each transcript. Protein level expression of T and CK-18 were examined by immunocytochemical method (Fig. 2E). Both proteins were clearly detected in both cultures, whilst T exclusively in cell nuclei and CK-18 in cytoplasm. The T and CK-18 positive cells each represented approximately 100% of all the examined population in both the contact and non-contact cultures (Fig. 2F). The total population was determined based on the DAPI staining. The result showed the generated cells are highly homogenous pertaining to the two typical notochordal markers.

### Influence of culture media

**Figure 3:**
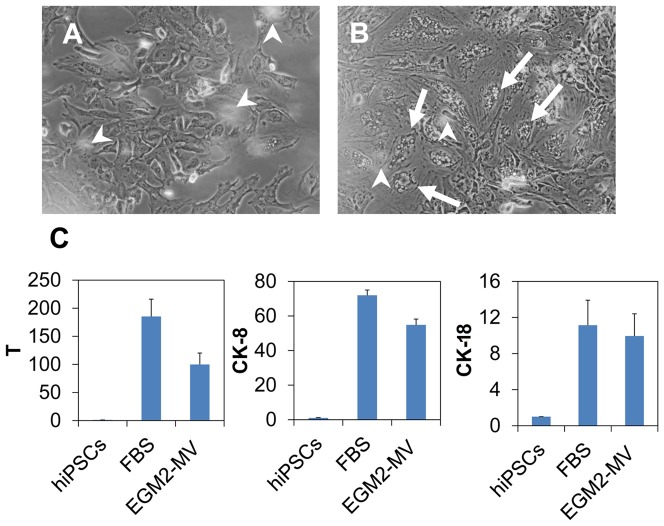
Influence of culture media on the notochordal differentiation. Two media was investigated: one contained 10% fetal bovine serum (FBS medium) and another contained a cocktail of growth factors (EGM2-MV medium, which is a commercial product of Lonza). (A) and (B) represents the cells differentiated for 5 days in FBS medium and EGM2-MV medium, respectively. Arrow heads indicate the floating NP tissue (out of focus in the image). Frequently the cells (arrows) in B displayed vacuole morphology which is also observable in primary culture of notochordal cells. (C): The transcript levels of three typical notochordal genes of the two cultures. The data are reported in relative mRNA expression which was analyzed by 2^−ΔΔCt^ method using hiPSCs as reference. Three biological samples were pooled and measured so that each result represents the average of three replicates.

The effect of two culture media was compared. In each culture medium, the porcine NP matrix was directly added (i.e., by the contact mode). One noticeable difference between the two cultures was the cell morphology (Fig. 3A, B). The cells in the Medium 1 (i.e., FBS medium) showed polymorphic shapes and a small portion of the cells showed a vacuole structure. In comparison, a large portion of the cells in the Medium 2 (i.e., EGM2-MV medium, Lonza) showed pronounced vacuole structure (Fig. 3B, arrows). Nevertheless, transcripts of the three notochordal marker genes (T, CK-8 and CK-18) were at comparable levels (Fig. 3C). Because of the similarity in gene expression, the two cells were putatively regarded comparable. Only the cells derived from the Medium 1 were further examined in the study.

### Functional differentiation to generate NP-like tissue

**Figure 4:**
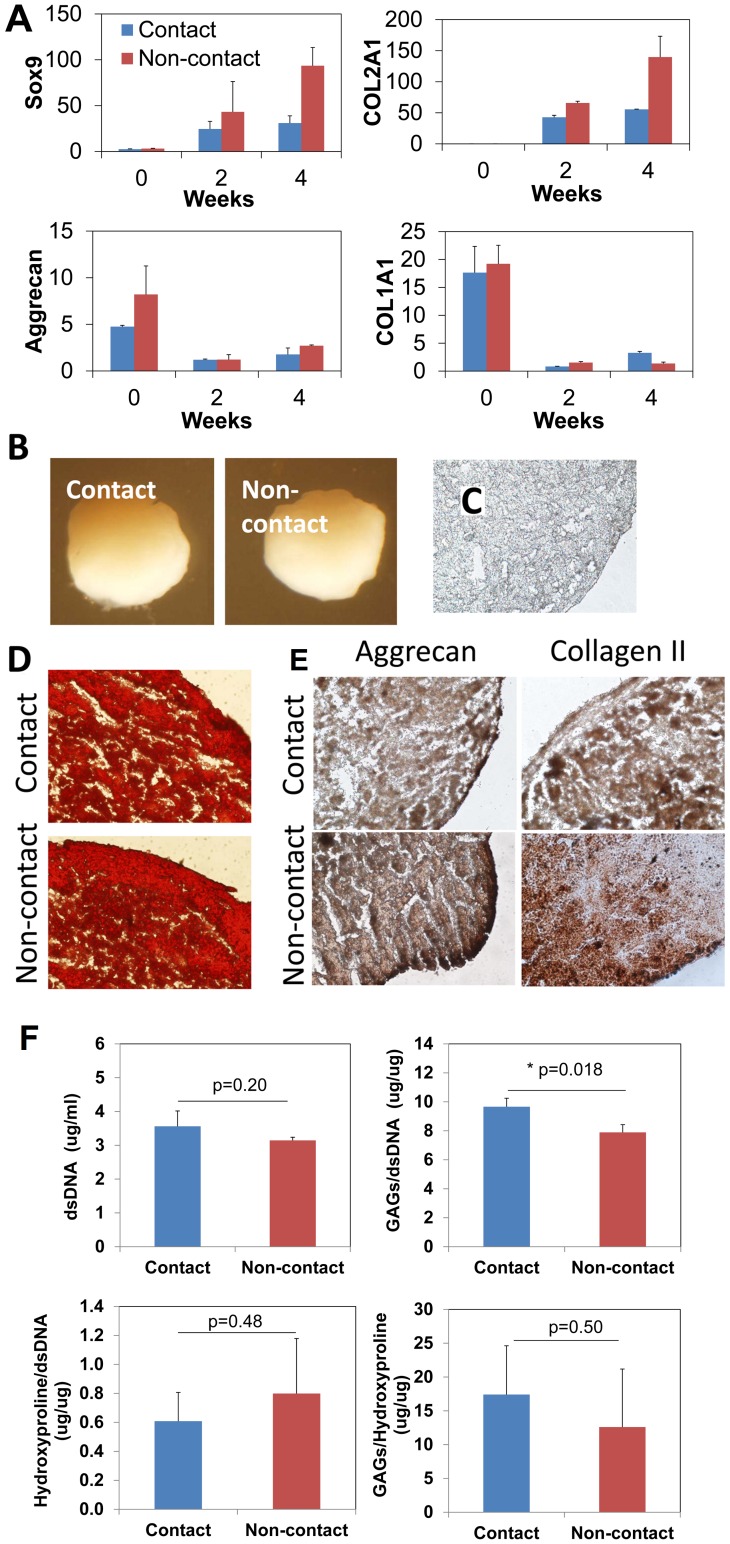
Functional differentiation of NCs to generate NP-like tissue induced by TGF-beta 3. The cultures were analyzed after 2 and 4 weeks. (A): The transcript of Sox9 and collagen type II (COL2A1) gene was remarkably up-regulated in both cells (NCs derived from contact or non-contact cultures) and at both time points. The transcript of aggrecan (ACAN) attenuated after 2 and 4 weeks comparing to the undifferentiated control (0-day), however, it is noteworthy that the ACNA transcript was maintained at significant levels in all the cultures (Ct∼30). The data are reported in relative mRNA expression which was analyzed by 2^−ΔΔCt^ method using day 0 control as reference. Three biological samples were pooled and measured so that each result represents the average of three replicates. The NC-like cells derived from both contact and non-contact cultures generated spherical balls with diameter of approximately 1 mm after 4 weeks (B). Extracellular biochemistry of the pellet cultures was analyzed after 4 weeks. Histological sections were cut at 5 um. (D): Safranin O staining of proteoglycans in the extracellular matrix. (E) represents immunohistochemical staining of aggrecan and collagen type II. Negative control (primary antibody omitted) was shown in (C). Magnification: (C, D and E): 400x. (F): Quantification of cell population (dsDNA content), glycosaminoglycans (GAGs) content, hydroxyproline content, and GAGs/hydroxyproline ratio of the NP differentiation cultures. The outcomes were compared between the cells derived from contact and non-contact cultures. Noticeably, the GAGs/hydroxyproline was as high as 12.6–17.4, which was remarkably higher than that of native hyaline cartilage (∼2), and much closer to that of native nucleus pulposus tissue (∼27). *: p<0.05, n = 3.

The generated NC-like cells were examined for the ability to terminally differentiate into NPCs and form NP tissue (Fig.4A–F). NPCs and NP tissue share similar phenotypes with chondrocytes and hyaline cartilage, but NP tissue distinguishes itself from cartilage with a high proteoglycan: collagen ratio (27∶1).[6], [24] [26] The NP differentiation can be stimulated by TGF-beta 3 following published protocols.[15], [22] The cells derived from both contact and non-contact culture modes were investigated.

Upon induction, three typical marker genes of NPC-like cells were detected including SOX9, aggrecan (ACAN), collagen type II (COL2A1) (Fig. 4A). Sox9 and collagen type II was remarkably up-regulated after 2 weeks and continued to increase up to 4 weeks. Aggrecan was also significantly detected (Ct: ∼30) at both time points, although the transcript levels were attenuated when compared to the undifferentiated zero-time control. Expression of collagen type I gene was also investigated as an adverse marker since it is minimally present in healthy NP tissue; it was actually down-regulated over the culture period directly contrasting to the concurrent up-regulation of collagen type II.

The NP-like tissues generated by both cell contact and non-contact were further examined on their ECM biochemistry. Both NC-like cells successfully formed spherical pellets (Fig. 4B). Histological sections were stained with Safranin O to identify proteoglycans. Figure 4D shows a strong and extensive staining of Safranin O on both cultures. Aggrecan (Fig. 4E) and collagen type II were detected by immunohistochemistry. Both molecules were clearly and extensively detected in both sections. The negative control did not show positive staining (Fig. 4C).

Proteoglycans and total collagen content in the NP-like tissues were quantified by biochemical methods (Fig. 4F). Proteoglycans were represented by the sulfated GAGs content; collagen was measured by quantifying hydroxyproline content after hydrolyzation of the pellets. The two cultures showed comparable cell population and hydroxyproline content after 4 weeks (p>0.05). The GAGs content was slightly higher (122%) in the tissue produced by the cells derived from the contact culture than the non-contact culture (p<0.05). The GAGs: hydroxyproline ratio was 17.4±7.2 and 12.6±8.6 (n = 3) for the tissues generated by NC-like cells derived from the contact and non-contact culture, respectively (p>0.05).

## Discussion

Cell therapy is an immerging technique for treating disc degeneration. Generating NCs from hiPSCs and other pluripotent stem cells will provide a stable, massive source for future routine clinical applications. Our study aims to develop a simple and effective method to generate NCs from hiPSCs. In the present work, we showed that direct contact between porcine NP tissue and hiPSCs is dispensable for the notochordal differentiation. This observation confirmed our speculation that the unknown inductive mediators (can be growth factors and other bioactive molecules) played a paracrine signaling role in the differentiation; the mediators are soluble and transferable to the hiPSCs through the culture medium. A similar mechanism was also demonstrated in a separate study, in which an enzyme-digested, solubilized extract of porcine NP tissue stimulated NP-like differentiation and matrix production of mesenchymal stem cells.[27] According to our present study, these unknown mediators can be spontaneously released from the NP matrix without the necessity of enzymatic solubilization. Corroborating with our observations, several other studies showed that living NCs can secrete certain agents into culture medium and stimulate NP differentiation of mesenchymal stem cells. [9], [22], [23] Distinguished from these previous studies, our work showed the NP matrix can induce the development of a NC phenotype rather than terminal NP phenotype.

The NC-like cells demonstrated excellent ability of functional differentiation to generate NP-like tissue in vitro. Cells derived from either contact or non-contact cultures showed a similar spectrum of transcript and protein expression of NP phenotypic markers, particularly, GAGs, aggrecan, and collagen type II. NP tissue resembles hyaline cartilage, and they share similar gene expression pattern and ECM biochemistry. [26], [28] An important characteristic that can distinguish them is the ratio of proteoglycan to collagen, which is high in native NP tissue (27∶1 of GAGs: hydroxyproline) but much low in hyaline cartilage (2∶1). [26] A high proteoglycan content is essential for the water-binding ability of native NP tissue and therefore the biomechanical functionality of IVDs. Loss of the proteoglycan content has direct implication of the development of disc degeneration. [1], [29] Our result clearly showed that aggrecan and GAGs were significantly expressed and deposited in the matrix. We noticed the aggrecan transcript decreased during the differentiation in comparison to the undifferentiated NC-like cells. Aggrecan is possibly regulated by a negative feedback loop, in which this core protein makes cells to attenuate its transcript; but it may not necessarily lower the protein production or comprise GAGs accumulation.[23] Immunohistochemical and biochemical and assays confirmed the significant deposition of aggrecan and GAGs in the matrix. Importantly, a high ratio of GAGs to hydroxyproline (in the range of 12.6 to 17.4) was detected which is much closer to that of native NP tissue rather than hyaline cartilage. Down-regulation of collagen type I corroborates the finding by indicating the unlikelihood of a fibrocartilage or fibrotic phenotype. Collectively, the results strongly suggest the differentiation was correctly directed to a NP phenotype but not a hyaline cartilage or other tissues. A limitation of the study is the potential hypertrophic differentiation was not examined. It possibly occurred since hypertrophy is commonly associated with in vitro chondrogenic [30], [31] and NP differentiation.[23] More appropriate culture condition, e.g., hypoxia,[32], [33] may avoid hypertrophy thus to help generate a healthy NC source. Future study will be conducted to systematically characterize the hypertrophic potential of the cells and optimize the differentiation condition.

In summary, we demonstrated the efficacy of generating NC-like cells from hiPSCs by harnessing the modulating effect of porcine NP tissue. The NP matrix can be added directly to the culture medium or separately via a culture insert. Direct contact between NP tissue and the cells may produce higher yield than the non-contact culture. The method is effective and reproducible and does not require additional growth factors and cytokines or cell sorting process. The generated NC-like cells displayed typical notochordal gene expression profile and functional differentiation ability to generate NP-like tissue in vitro. In particular, the generated NP-like tissue had a high GAGs: hydroxyproline ratio which is close to that of native NP tissue. This study corroborated our previous findings, and it provided a solid foundation for future more comprehensive studies on the biology of the NC-like cells and using the cells to treat degenerative IVDs.
